# Vascular Endothelial Growth Factor-B Induces a Distinct Electrophysiological Phenotype in Mouse Heart

**DOI:** 10.3389/fphys.2017.00373

**Published:** 2017-05-31

**Authors:** Nikolay Naumenko, Jenni Huusko, Tomi Tuomainen, Jussi T. Koivumäki, Mari Merentie, Erika Gurzeler, Kari Alitalo, Riikka Kivelä, Seppo Ylä-Herttuala, Pasi Tavi

**Affiliations:** ^1^A.I. Virtanen Institute for Molecular Sciences, University of Eastern FinlandKuopio, Finland; ^2^Wihuri Research Institute and Translational Cancer Biology Program, University of Helsinki, Biomedicum HelsinkiHelsinki, Finland; ^3^Heart Center and Gene Therapy Unit, Kuopio University HospitalKuopio, Finland

**Keywords:** cardiac myocytes, growth factors, EC-coupling, energy metabolism, ion channels, action potential

## Abstract

Vascular endothelial growth factor B (VEGF-B) is a potent mediator of vascular, metabolic, growth, and stress responses in the heart, but the effects on cardiac muscle and cardiomyocyte function are not known. The purpose of this study was to assess the effects of VEGF-B on the energy metabolism, contractile, and electrophysiological properties of mouse cardiac muscle and cardiac muscle cells. *In vivo* and *ex vivo* analysis of cardiac-specific VEGF-B TG mice indicated that the contractile function of the TG hearts was normal. Neither the oxidative metabolism of isolated TG cardiomyocytes nor their energy substrate preference showed any difference to WT cardiomyocytes. Similarly, myocyte Ca^2+^ signaling showed only minor changes compared to WT myocytes. However, VEGF-B overexpression induced a distinct electrophysiological phenotype characterized by ECG changes such as an increase in QRSp time and decreases in S and R amplitudes. At the level of isolated TG cardiomyocytes, these changes were accompanied with decreased action potential upstroke velocity and increased duration (APD_60–70_). These changes were partly caused by downregulation of sodium current (I_Na_) due to reduced expression of Na_v_1.5. Furthermore, TG myocytes had alterations in voltage-gated K^+^ currents, namely decreased density of transient outward current (I_to_) and total K^+^ current (I_peak_). At the level of transcription, these were accompanied by downregulation of K_v_ channel-interacting protein 2 (*Kcnip2*), a known modulatory subunit for K_v_4.2/3 channel. Cardiac VEGF-B overexpression induces a distinct electrophysiological phenotype including remodeling of cardiomyocyte ion currents, which in turn induce changes in action potential waveform and ECG.

## Introduction

The Vascular Endothelial Growth Factor (VEGF) family consists of five proteins with regulative roles in blood and lymphatic vessel growth and development (Bry et al., 2014). The angiogenic potency of VEGFs makes them attractive candidates for therapy of ischemic heart (Vuorio et al., 2012). However, among the VEGFs, the effects of VEGF-B spread beyond vessel regulation as it has been implicated in the regulation of myocardial metabolism, growth, and stress-response (Karpanen et al., 2008; Huusko et al., 2012; Kivela et al., 2014). VEGF-B is expressed in a variety of tissues with highest expression in heart and oxidative skeletal muscle (Hagberg et al., 2010). Alternative splicing of the VEGF-B gene produces two isoforms, VEGF-B_167_ and VEGF-B_186_, which both mediate their effects by binding to VEGFR-1 (Olofsson et al., 1998) and NRP-1 receptors (Makinen et al., 1999). When overexpressed, VEGF-B activates Akt/mTORC1 and ERK1/2 MAPK pathways (Kivela et al., 2014). Data from genetic rodent models suggest that VEGF-B is not a prerequisite for cardiac development or function, but rather serves as a regulatory factor. Supporting this, VEGF-B deficient mice are viable and show only mild cardiac phenotype with either atrial conduction defect (Aase et al., 2001) or retarded growth accompanied with slightly impaired coronary vasculature and reduced ischemic tolerance *ex vivo* (Bellomo et al., 2000). Similarly, VEGF-B deficient rats have no obvious abnormalities in either contractile or vascular function (Kivela et al., 2014). Transgenic overexpression of VEGF-B in mouse (Karpanen et al., 2008) or rat heart (Bry et al., 2010; Kivela et al., 2014) induces metabolic remodeling, moderate hypertrophy, and myocardial capillary enlargement with improved ischemic tolerance. VEGF-B delivery into myocardium with adenoviruses in rodent or pig hearts consistently produces an increase in the density or diameter of the myocardial capillaries, and AAV-VEGF-B delivery appears to protect the heart against ischemia-, pacing-, or load-induced cardiomyopathy (Bry et al., 2014). So far, studies on cardiac effects of VEGF-B have been mainly conducted in the context of angiogenic therapy to enhance perfusion of the heart and metabolic effects on the myocardium. However, the effects of VEGF-B on myocardial function at the level of cardiac muscle cells are not known. The purpose of this study was to assess the effects of VEGF-B on the energy metabolism, contractile, and electrophysiological properties of the mouse heart.

## Materials and methods

### Experimental animals

The present studies employed 2–4 months old transgenic (TG) mice overexpressing human VEGF-B_167_ and VEGF-B_186_ isoforms (Supplementary Figure S1) under the cardiac-specific myosin heavy chain promoter (Bry et al., 2010). VEGF-B TG mice were bred into the C57Bl/6JOlaHsd background for over 10 generations. TG-negative littermates (WT) were used as controls in all experiments. The animals were kept in standard housing conditions in The National Laboratory Animal Center of The University of Eastern Finland. Diet and water were provided *ad libitum*. All animal experiments were performed according the guidelines of Directive 2010/63/EU of the European Parliament on the protection of animals used for scientific purposes, the procedures were also approved by the Animal Experiment Board in Finland and carried out in accordance with the guidelines of the Experimental Animal Committee of the University of Eastern Finland.

### Echocardiography and electrocardiography

Ten TG and ten WT littermate controls were used for echocardiographic and electrocardiographic measurements performed with the Vevo2100 Ultrasound System (VisualSonics Inc., Toronto, ON, Canada). A high-frequency ultrasound probe (MS400) operating at 30 MHz with a minimum of 300 frames per second was used. The animals were anesthetized with isoflurane (induction: 4.5% isoflurane, 450 mL air, maintenance: 2.0% isoflurane, 200 mL air, Baxter International Inc., Deerfield, IL, USA). Mice were placed in a supine position on a heated platform (THM100, Indus Instruments, Houston, TX, USA) to maintain the body temperature at 36–37°C. Ejection fraction (EF), left ventricle anterior wall thickness (LVAW), left ventricle volume (LV Vol), and left ventricle mass (LV Mass) were determined from parasternal short axis M-Mode measurements. EF was calculated by Vevo2100 software using the Teicholz formula. To obtain the ECG signal, the paws of the mice were connected to the electrode pads on the platform using ECG gel and fixed with a skin tape. The recorded ECG represents the standard limb lead II. The mouse heart rate and respiration were monitored during anesthesia via ECG pads. The raw data of ECG were analyzed with a Matlab-based ECG analysis program (Kubios HRV, version 2.0 beta 4, Department of Physics, University of Eastern Finland, Kuopio, Finland), which was modified specially for analyzing mouse ECG (Merentie et al., 2015). Time intervals (QRS, QRSp, and QTc time) and amplitudes of R and S wave were analyzed from the mean curve generated from a 30-s ECG recording. QTc time was calculated as QT ms/(R − R_0_/100 ms)^1/2^ (Mitchell et al., 1998).

### Langendorff heart perfusion model

For *ex vivo* heart studies, conventional Langendorff perfusion was used (Bell et al., 2011). Isolated hearts were perfused with 37°C Krebs-Henzeleit buffer solution and paced by surface electrodes at 8 Hz in all experiments. To record left ventricular pressure (LVP), a water-filled PVC balloon was inserted into the left ventricle (LV). Heart rate and LVP were monitored with an HSE ISOTECH Pressure Transducer and recorded with a data acquisition board (PCI-6052E, National Instruments, Austin, TX, USA). Collected data were analyzed with WinEDR software (University of Strathclyde, Glasgow, UK). To assess the substrate specificity (glucose vs. palmitate) of the isolated hearts, the buffer solution was supplemented with 1.2 mM sodium palmitate (Sigma-Aldrich, St. Louis, MO, USA) conjugated to fatty acid-free bovine serum albumin (BSA, Sigma-Aldrich, St. Louis, MO, USA) (Belke et al., 1999) or with 11 mM glucose and unconjugated BSA.

### Single cell isolation

Adult mouse ventricular myocytes were obtained by enzymatic dissociation as described before (AfCS Procedure Protocol PP00000125). Briefly, isolated hearts were placed in a Langendorff apparatus for perfusion (37°C, 3 mL/min) with a trypsin (Sigma) and liberase (Roche Applied Science) solution. After that, ventricles were cut into small pieces and gently minced with a Pasteur pipette. The concentration of Ca^2+^ in solution was increased slowly up to 1 mM. The suspension of cells was placed on laminin-coated coverslips and stored in an incubator (5% CO_2_ at 37°C).

### Size measurement of the isolated cardiomyocytes

Adult cardiomyocyte size was assessed with a Coulter Counter Z2 (Beckman Coulter) equipped with a 200 μm aperture and data analyzed with Accucomp software (Beckman Coulter). 20% of the isolated cells from one heart were suspended in 2 mL medium and 10 mL Beckman Isoton II diluent (Beckman Coulter) was added. The metered volume was 1 mL, main gain 32 and aperture current 0.250. From each analysis mean and median cell volumes of the cell population above the size of 20,000 fL were recorded. The cell population below 20,000 fL (equal to a spherical particle with 17 μm diameter) was contaminated with smaller cells and cell debris, and therefore discarded from the analysis (Supplementary Figure S2).

### Western blot

Frozen ventricular tissue was lysed with a TissueLyser II (Qiagen) and protein concentration of the lysate was measured with the Bio-Rad Protein Assay. Ten to fifty micrograms of protein was loaded onto an 8–12% SDS polyacrylamide gel. After gel electrophoresis, proteins were transferred to a nitrocellulose membrane (0.2 μm, Bio-Rad Laboratories), which was blocked with 5% BSA. For blotting of the large (240 kDa) Na_v_1.5 protein, a gradient gel (4–15%, Bio-Rad Laboratories, #456–1,083) was used and protein samples transferred to a 0.4 μm membrane. Membranes were incubated with primary antibody, after which incubation with Cy5 (GE Healthcare, PA45012) or Cy3 (PA43010)-linked secondary antibody was performed and blots visualized with the ECL Plus Detection Kit (GE Healthcare) using a Typhoon 9400 scanner (GE Healthcare). The antibodies used were: Anti-Na_v_1.5 (Alomone labs, ASC-005), Anti-VEGF-B (R&D Systems, Af751), and β-actin (Cell Signaling Technology, 4967). Ponceau S staining solution (Sigma-Aldrich, St. Louis, MO, USA) was used as per manufacturer's instructions to visualize total membrane protein as a loading control for the blotted Na_v_1.5 protein.

### Metabolic analysis of isolated cardiomyocytes

To assess mitochondrial respiration, a Seahorse XF24 Analyzer (Seahorse Bioscience, North Billerica, MA, USA) was used as described in the Online Data Supplement. To assess the substrate specificity (glucose vs. palmitate) of the isolated cardiomyocytes, the assay buffer was supplemented with 200 μM sodium palmitate (Sigma-Aldrich, St. Louis, MO, USA) conjugated to fatty acid-free bovine serum albumin (BSA, Sigma-Aldrich, St. Louis, MO, USA; Belke et al., 1999) or with 25 mM glucose and unconjugated BSA.

### Gene expression

Primers and probes designed to detect mouse mRNA transcripts in Taqman-based RT-qPCR are listed in Supplementary Table S1.

### Patch-clamp experiments

For individual currents or action potential measurements, an Axopatch-200B amplifier and Digidata 1440A A/D-D/A and Clampex 10 software (Axon Instruments, Sunnyvale, CA, USA) were used as previously (Karppinen et al., 2016). Previously described solutions and protocols were used for L-type Ca^2+^ current (Xu et al., 2011), K^+^ currents (Rivard et al., 2009), Na^+^-currents (Sato et al., 2009; Sossalla et al., 2010), and for action potential measurements (Yang et al., 2005). Late I_Na_ was estimated as integration of the current from 50 to 250 ms of the beginning of the depolarizing pulse (250 ms) without and with ranolazine (10 μM) in the bath solution. Current-voltage relations for steady-state activation and inactivation for calcium and sodium currents were determined by fitting a Boltzmann function (*I/I*_max_ = [1 + exp ((*V* − *V1/2*)/*k*)]^−1^), yielding the membrane potential of the half-maximal activation (*V1/2*-activation) and inactivation (*V1/2*-inactivation) and slope factor (*k*). The current recovery from inactivation curves were fitted using a single exponential function. Data analysis was made using Clampfit10 software (Molecular Devices Inc., USA).

### Ca^2+^ measurements

Adult cardiomyocytes were loaded with Fluo-4-acetoxymethyl (AM)-ester (10 μM; 0.02% pluronic acid, Invitrogen, Carlsbad, CA, USA), calcium signals were recorded with a confocal microscope, and data were analyzed as previously (Tavi et al., 2005; Korhonen et al., 2010).

### Computer modeling

Some of the *in vitro* experiments were replicated *in silico* to evaluate the contribution of individual VEGF-B related changes on AP morphology, using mathematical model of mouse ventricular myocyte (Koivumaki et al., 2009). The VEGF-B related modifications were implemented to mutant model variants based on experimental data, details are available in the Supplementary Methods.

### Statistical testing

Data and statistical analyses were made using Origin9 software (OriginLab Corp., Northampton, MA, USA). For statistical analysis, one-way ANOVA with Fisher's *post-hoc* comparison was performed for all ion currents and calcium transient frequency dependence, as well as Student's *t*-tests where appropriate, were applied at a level of significance of *P* < 0.05. Data are given as mean values ± SEM.

## Results

### VEGF-B mice display normal heart function and cardiomyocyte hypertrophy without metabolic changes

**Figure 1:**
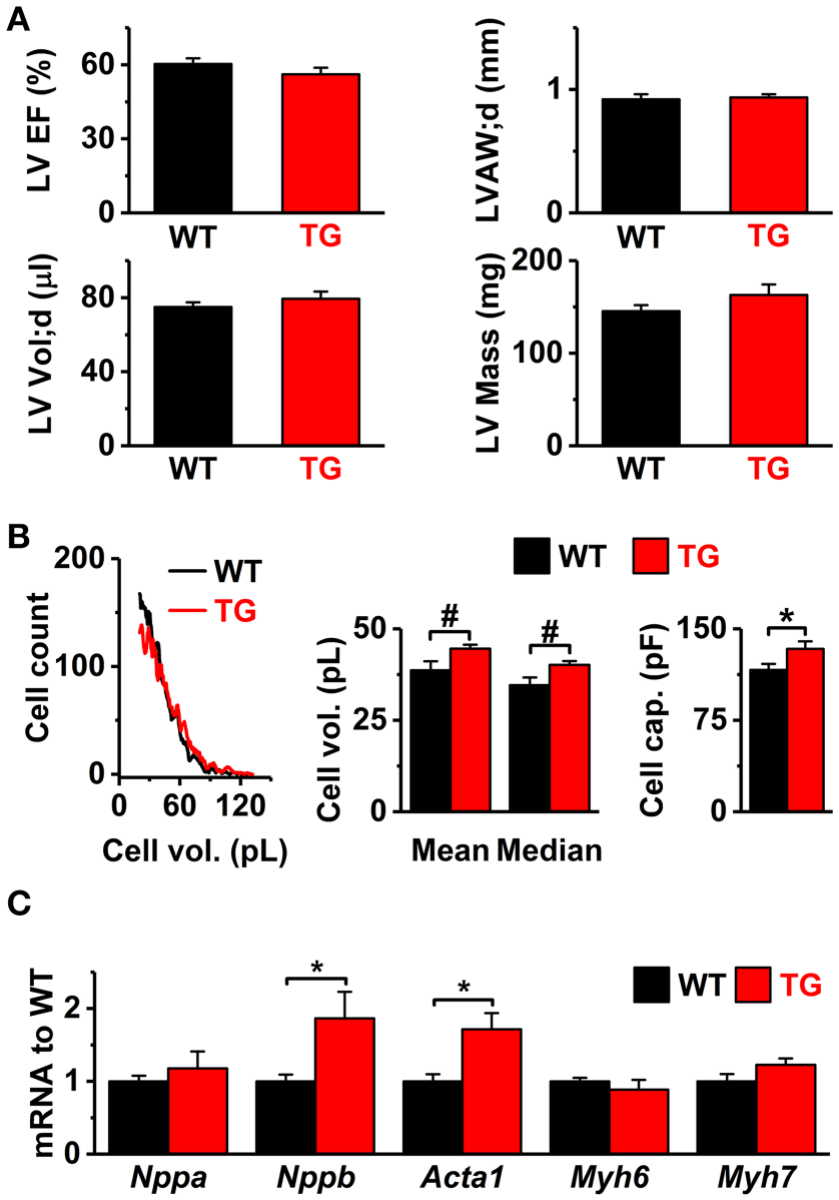
*In vivo* cardiac function, cell size, and gene expression of hypertrophy markers in VEGF-B mice. **(A)** *In vivo* echocardiography data of left ventricle (LV): ejection fraction (LV EF%, upper left), diastolic anterior wall thickness (LVAW;d, upper right), diastolic volume (LV Vol;d, lower left), and mass (LV Mass, lower right, *n* = 10 for both groups). **(B)** Size of the isolated cardiomyocytes. Left panel: representative Coulter Counter histograms. Middle panel: average of means and medians from populations of cells isolated from hearts (WT; six hearts, 4152 ± 1770 cells/analysis TG; seven hearts, 6,040 ± 1,533 cells/analysis). Right panel: means of cell capacitances measured in whole-cell experiments (WT *n* = 8/51 and TG *n* = 10/58; animals/cells). **(C)** Gene markers of cardiac hypertrophy [mRNA to WT ratio: natriuretic peptide A (*Nppa*), natriuretic peptide B (*Nppb*), skeletal actin (*Acta1*), myosin heavy polypeptide 6 (*Myh6*), and myosin heavy polypeptide 7 (*Myh7*); WT *n* = 7 and TG *n* = 6]. ^*^P < 0.05, #*P* < 0.001.

**Figure 2:**
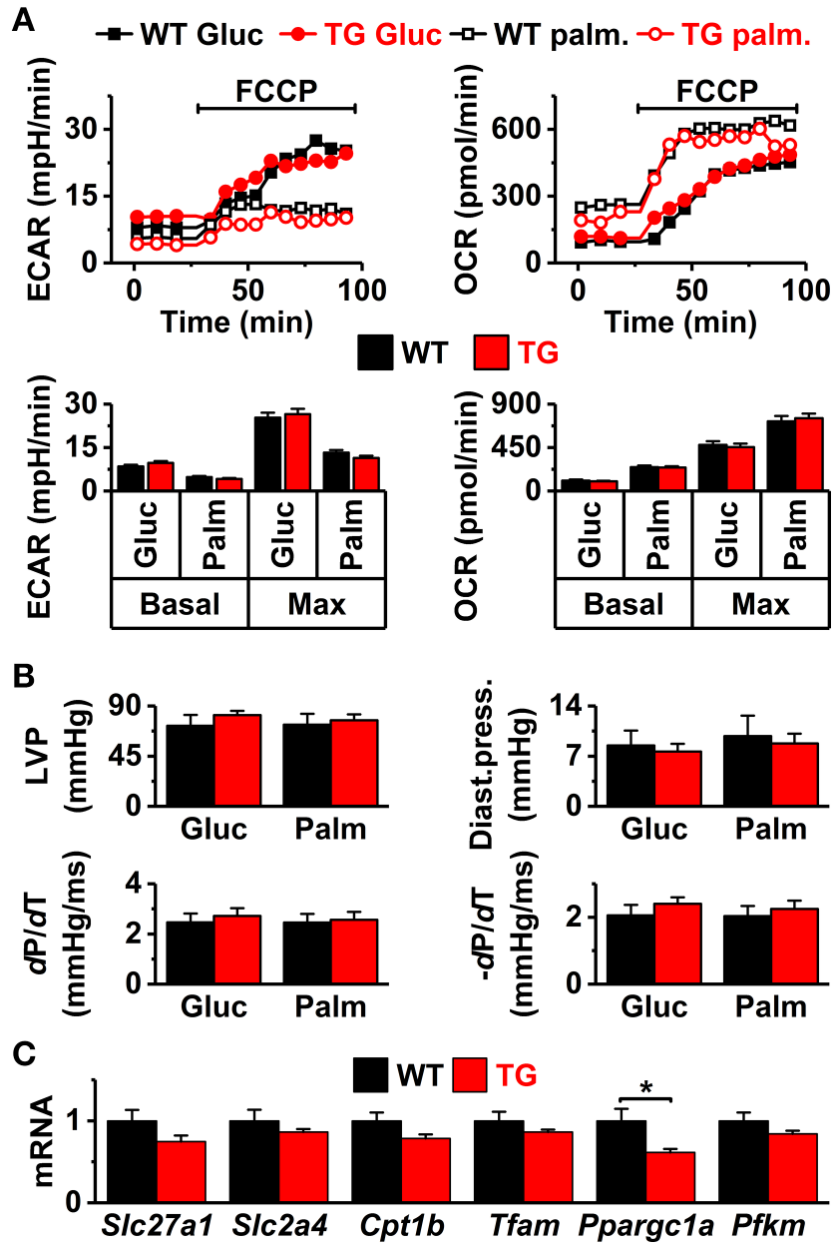
Cell energy metabolism and *ex vivo* cardiac function. **(A)** Extracellular acidification and oxygen consumption in isolated cardiomyocytes supplemented with either glucose (Gluc) or palmitate (Palm). Top panels: representative plots of extracellular acidification rate (ECAR, left) and oxygen consumption rate (OCR, right). Bottom panels: basal and FCCP-induced maximal ECAR (left) and OCR [right, *n* = 35 wells with ~2,000 cells each, from seven (7 WT and 7 TG mice) independent paired assays]. **(B)** *Ex vivo* systolic heart function in glucose- (Gluc) and palmitate-based (Palm) solutions. Systolic pressure (LVP, upper left), diastolic pressure (Diast.press., upper right), systole rise slope (dP/dT, lower left), and systole decay slope (−dP/dT, lower right, *n* = 6 in both groups). **(C)** Ventricular expression of the genes (normalized to WT) related to energy metabolism: fatty acid transporter (*Slc27a1*), glucose transporter (*Slc2a4*), carnitine palmitoyltransferase 1B (*Cpt1b*), mitochondrial transcription factor A (*Tfam*), muscle phosphofructokinase (*Pfkm*), and peroxisome proliferator-activated receptor gamma coactivator 1-alpha (*Ppargc1a*) (WT *n* = 7 and TG *n* = 6) ^*^P < 0.05.

VEGF-B has been shown to induce multiple adaptations in the heart, including angiogenesis and muscle growth (Bry et al., 2014). According to echocardiographic analysis, the VEGF-B mice had normal systolic and diastolic heart function and mild signs of cardiac hypertrophy (Figure 1). However, there was a significant increase in the volume and cell capacitance of isolated VEGF-B cardiomyocytes (Figure 1B, Supplementary Figure S2). This was accompanied with increased expression of the hypertrophy markers *Nppb* and *Acta1* (Figure 1C), indicating an induction of hypertrophic pathways. Cardiac VEGF-B overexpression in rats has been shown to induce metabolic changes with decreased expression of fatty acid oxidation genes and increased concentration of intermediates of glucose metabolism (Kivela et al., 2014). We therefore assessed the oxidative and anaerobic metabolism and substrate preference of isolated cardiomyocytes with a Seahorse extracellular flux analyser. Extracellular acidification (ECAR), which reflects glycolysis rate, did not differ between the TG and WT cardiomyocytes and neither was there a difference in oxygen consumption (OCR) between the groups (Figure 2A). Moreover, we did not find changes in the energy substrate preferences; basal and maximal ECAR and OCR were similar in WT and TG animals when the energy source was either glucose or fatty acid (palmitate, Figure 2A). To assess the effect of energy substrate on the contractile function of the heart, we fed Langendorff-perfused hearts either glucose or palmitate and measured LV function. We saw no difference between TG and WT LV function with either glucose or palmitate (Figure 2B). In line with these findings, expression of genes related to cellular and mitochondrial energy metabolism was similar in TG and WT hearts (Figure 2C). Collectively, our data suggest that in isolated TG cardiomyocytes, changes in energy metabolism are not evident. Furthermore, the VEGF-B induced metabolic changes do not involve changes in substrate uptake, as suggested earlier (Kivela et al., 2014).

### VEGF-B overexpression augments Ca^2+^ signals in ventricular myocytes

**Figure 3:**
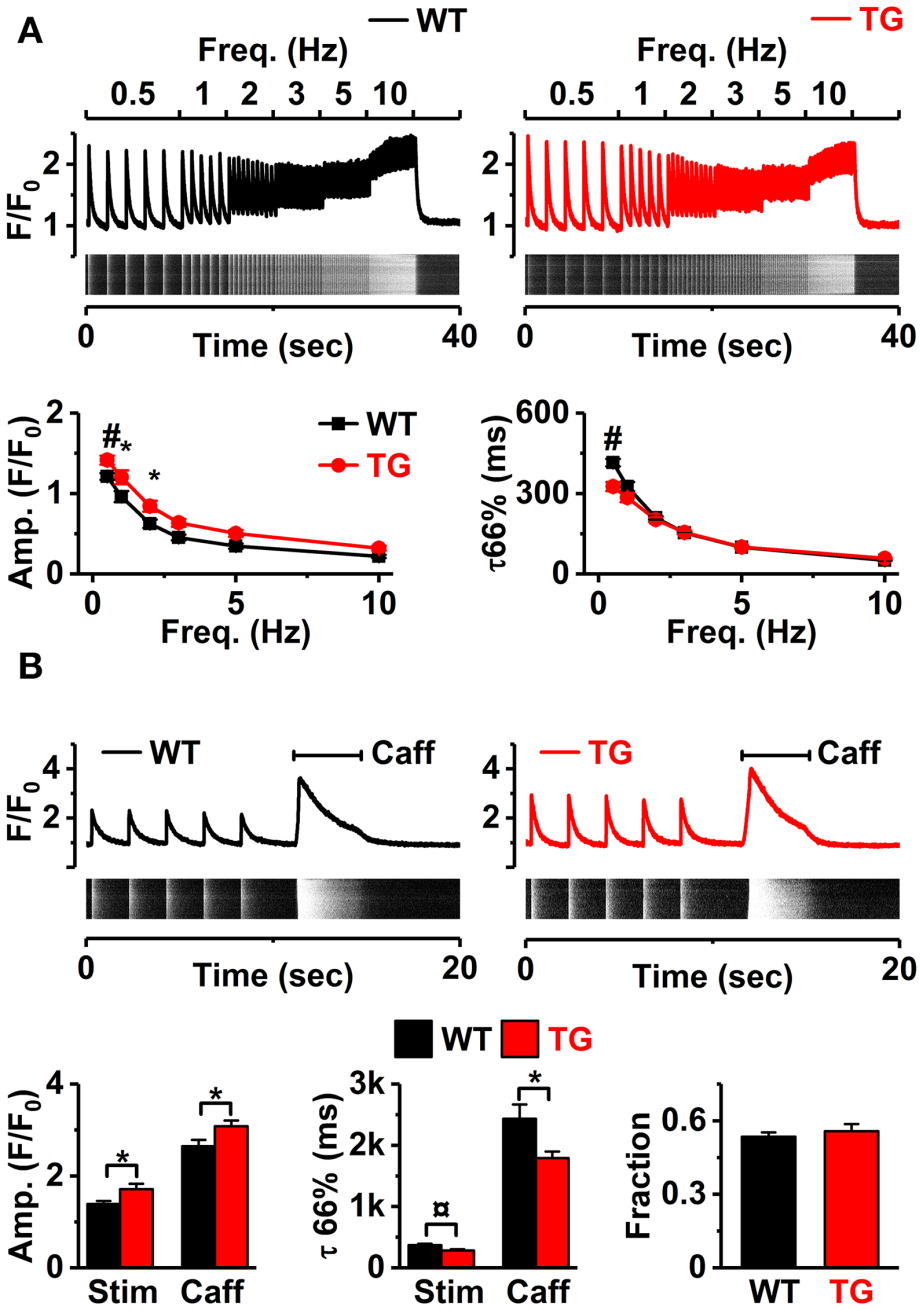
Ca^2+^ handling in VEGF-B TG ventricular myocytes. **(A)** Representative [Ca^2+^]_i_ transients (line-scans) and their profiles in Fluo4-loaded cardiomyocytes upon pacing at different stimulus frequencies in WT and TG mice (upper), frequency dependence of amplitudes (lower left), and time constants (lower right) of the [Ca^2+^]_i_ transients (*n* = 16 myocytes for WT and *n* = 22 myocytes for TG, at least three animals were used for both groups). **(B)** Representative line-scans and profiles of stimulus- and caffeine-induced [Ca^2+^]_i_ pulses in WT (upper left) and TG mice (upper right). Amplitudes (lower left) and time constant of decays (lower middle) of stimulus- (Stim) and caffeine-induced (Caff) transients, and fractional release (lower right, WT *n* = 23 myocytes and TG *n* = 26 myocytes, at least three animals were used for both groups). ^*^P < 0.05, ¤*P* < 0.01 and #*P* < 0.001.

Next, we studied if VEGF-B overexpression affects the [Ca^2+^]_i_ signaling of the myocytes. To challenge the cells, we first stimulated Fluo-4-loaded isolated cardiomyocytes at different frequencies (0.5–10 Hz, Figure 3A). The amplitude of [Ca^2+^]_i_ transients was higher in TG cardiomyocytes at low stimulus frequencies (0.5–2 Hz, Figure 3A). The time constant of [Ca^2+^]_i_ transient decay, which indicates calcium extrusion rate, was shorter but only at the lowest pacing frequency (0.5 Hz, Figure 3A). SR Ca^2+^ loading, estimated by rapid application of caffeine (10 mM; Figure 3B) was higher in TG myocytes (Figure 3B). While VEGF-B TG myocytes showed increased [Ca^2+^]_i_ transient amplitude and SR-load, the fractional release, assessed by the ratio of stimulus- and caffeine-induced [Ca^2+^]_i_ transients, was not altered in TG vs. WT myocytes. These changes were accompanied with downregulation of *Atp2a2* (*Serca2a)* expression, while *Atp2a2*/phospholamban ratio was not altered (Supplementary Figure S3B). Moreover, caffeine induced [Ca^2+^]_i_ transient was shorter in TG animals (Figure 3B) which could reflect increased I_NCX_. Taken together, although VEGF-B TG myocytes show augmented calcium transients (CaTs) and increased SR load, these changes are not due to extensive remodeling and are likely to have a relatively small impact on cardiomyocyte function at physiological heart rates *in vivo*.

### VEGF-B induces alterations of electrophysiological properties of the mouse heart

**Figure 4:**
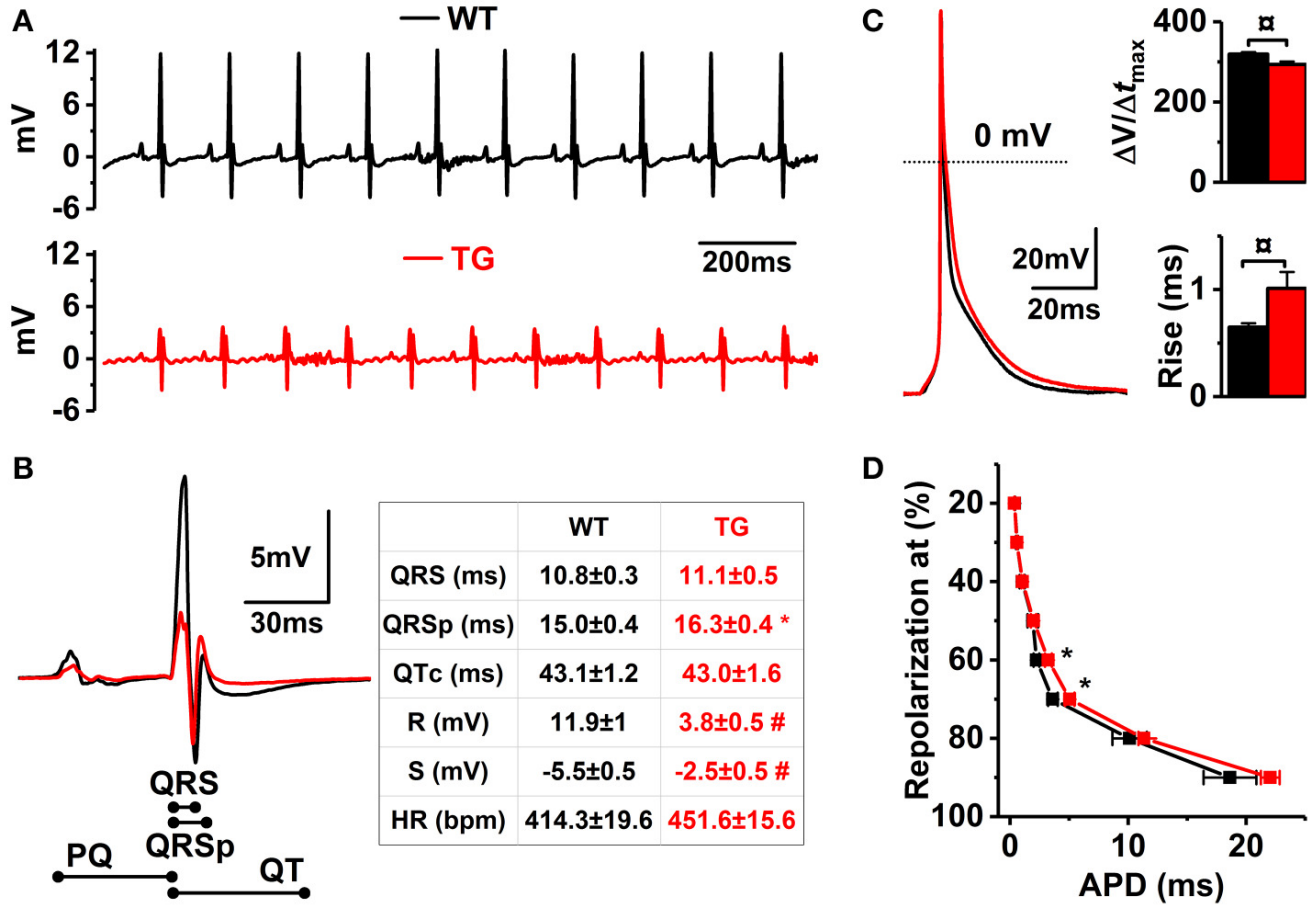
Electrophysiological properties of VEGF-B TG hearts and isolated cardiomyocytes. **(A)** Representative ECG recordings from WT (black line) and VEGF-B TG (red line) mice. **(B)** Enlarged superimposed ECG complexes (left panel) with summary of the ECG parameters (right panel, *n* = 10 for both groups). **(C)** Representative superimposed AP traces from isolated myocytes (left) and means of the AP maximum rise slope (upper right) and rise time (lower right). **(D)** AP durations at different percentages of repolarization (WT *n* = 17 and TG *n* = 34; 4 animals for both group). ^*^P < 0.05, ¤*P* < 0.01 and #*P* < 0.001.

VEGF-B deficiency has been shown to interfere with atrial conduction in mice (Aase et al., 2001), indicating that VEGF-B regulates mechanisms maintaining electrophysiological properties of the heart. Thus, VEGF-B overexpression might result in remodeling of electrical properties of myocardium. Comparison of the ECGs of WT and VEGF-B TG mice indicated drastic changes in the ECG waveform of TG hearts (Figures 4A,B). Specifically, TG hearts had decreased amplitudes of R- and S-waves accompanied by an increase in the QRSp time which reveals early repolarization in the heart (Figure 4B), whereas QRS width was not changed. ECG changes, such as those seen in VEGF-B TG hearts, can be induced by changes in the electrical properties of myocytes or passive properties of the heart tissue (Dhein et al., 2014). VEGF-B TG hearts had no signs of fibrosis based on LV relaxation (Figure 2B) and histology (data not shown) or changes in the expression patterns of connexin isoforms (Supplementary Figure S3A), but isolated ventricular myocytes had significant changes in action potential (AP) waveform. However, TG myocytes had no significant changes in APD_90_ they had increased duration of AP at 60 and 70% of repolarization (~40 and ~45% in APD_60_ and APD_70_, respectively; Figures 4C,D). Furthermore, TG myocytes had an increase in the rise time and slowing of maximum rise slope of the AP upstroke (Figure 4C). These results suggest that ECG changes are due to remodeling of the electrical properties of the myocytes involving both depolarizing and repolarizing currents.

### VEGF-B TG myocytes have depressed I_Na_

**Figure 5:**
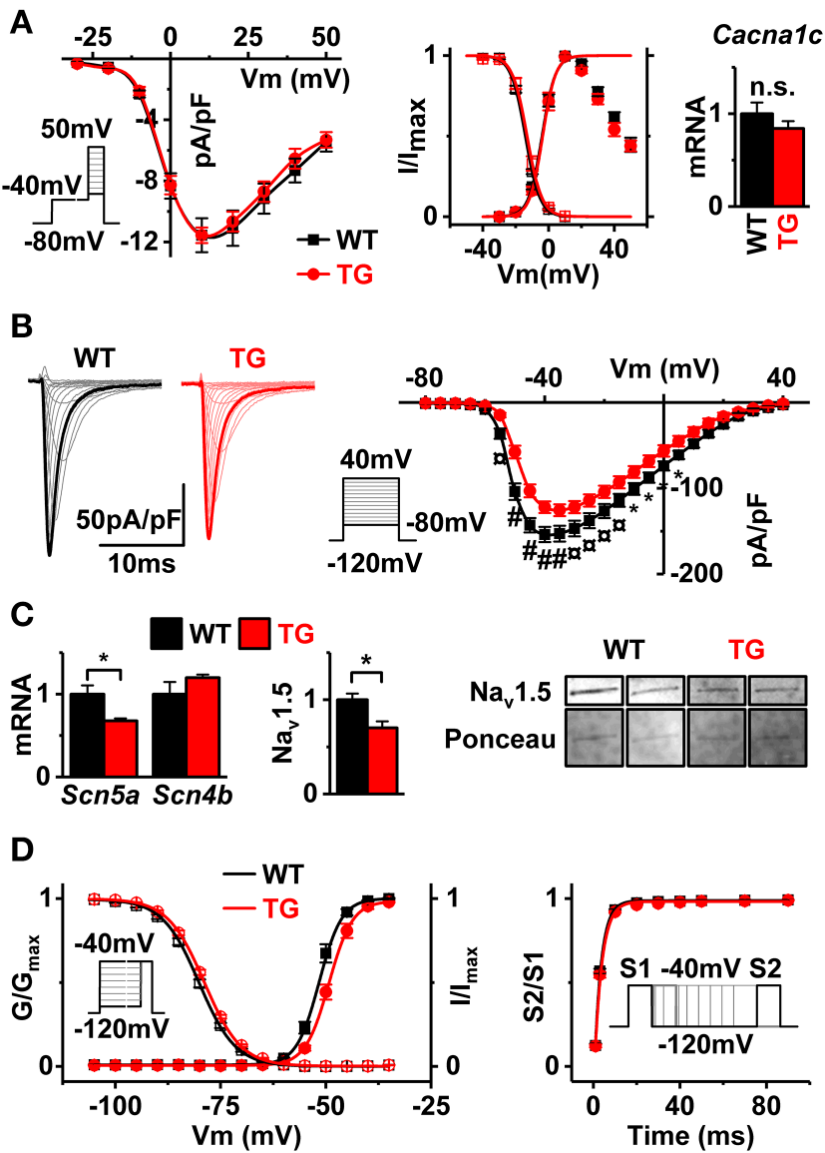
Depolarizing currents in VEGF-B TG myocytes. **(A)** I–V curves of I_CaL_ densities (left panel, WT *n* = 9 and TG *n* = 11, myocytes from three animals in both groups), voltage-dependent activation/inactivation of I_CaL_ (middle panel, Boltzmann fitting) and gene expression of α-subunit calcium channel (*Cacna1c*, right panel). **(B)** Typical sodium channel current traces (left panel) and I–V curves (right panel) (WT black line, *n* = 19 and TG red line *n* = 14 myocytes from 3 mice were used for both groups). **(C)** Gene expression of sodium channel subunits *Scn5a* and *Scn4b* (left, WT *n* = 7 and TG *n* = 6) and Western blot of Na_v_1.5 from membrane protein fraction. Na_v_1.5 western blot signal normalized to Ponceau stain (middle, WT *n* = 3, TG *n* = 4). Representative SCN5a bands and Ponceau stain from the same region of the membrane (right). **(D)** Voltage-dependent activation and inactivation of sodium channels (left panel) and recovery time from inactivation (right panel) (see Table S3 for fitting results, WT *n* = 19 myocytes and TG *n* = 15 myocytes from three animals in both groups). Refer to insets for voltage-step protocols. ^*^P < 0.05, ¤*P* < 0.01 and #*P* < 0.001.

To specify the ion current changes causing ECG and AP changes in VEGF-B TG hearts, we first measured L-type Ca^2+^ channel current (I_CaL_) from cardiomyocytes isolated from TG and WT hearts. We saw no differences in the I–V-dependence, density, activation/inactivation, or expression of the *Cacna1c* gene encoding the pore-forming subunit of the L-type channel (Figure 5A, Supplementary Table S2). In contrast to I_CaL_ the other major depolarizing current, I_Na_ was significantly decreased in VEGF-B TG myocytes (Figure 5B). The decrease of the I_Na_ density was accompanied with downregulation of the *Scn5a* mRNA and Na_v_1.5 protein (Figure 5C). Moreover, I_Na_ from VEGF-B TG myocytes showed a rightward shift in both voltage-dependent activation and inactivation (Figure 5D, Supplementary Table S3) without a change in recovery time after inactivation (Figure 5D, Supplementary Table S3). Another property of Na-channels subject to modifications is the slowly inactivating component (Gintant et al., 1984), also known as the late Na-current. Since the late I_Na_ has been linked to ECG changes such as those seen in VEGF-B mice (Moreno and Clancy, 2012), we assessed the I_Na-L_ in VEGF-B TG myocytes. However, we did not find differences in late I_Na_ between WT and TG myocytes either with I_Na-L_ blocker ranolazine or by a voltage clamp protocol specific for late Na^+^-current even in condition when physiological [Na^+^]_out_ concentration was used (Supplementary Figure S4). Altogether, decreased I_Na_ gives a reasonable explanation for the ECG anomalies (Figures 4A,B) and decrease in the AP upstroke velocity (Figure 4C).

### VEGF-B affects the expression and density of potassium currents

**Figure 6:**
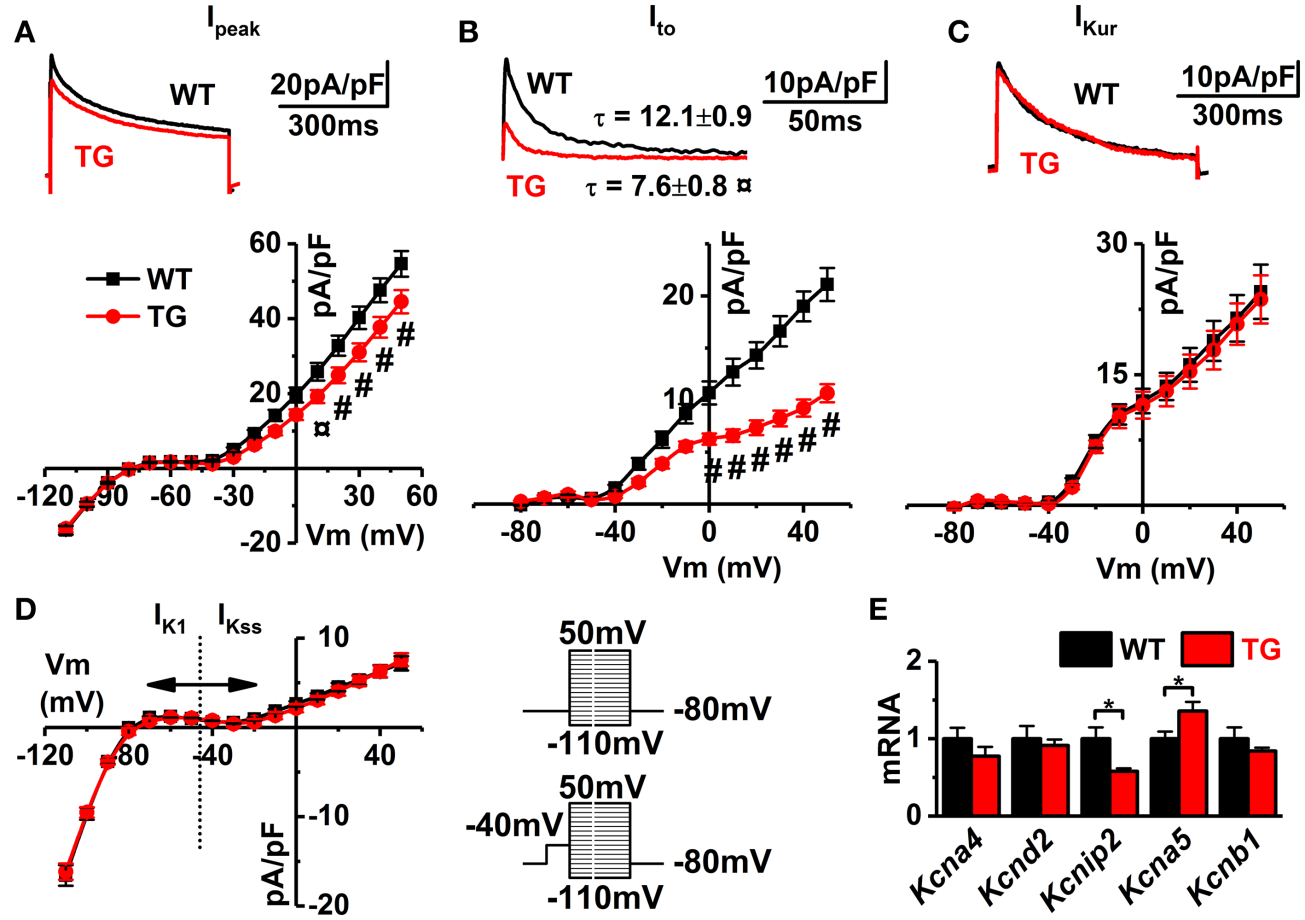
Voltage-gated potassium currents. Representative superimposed original traces (upper, obtained at + 50 mV) and I–V curves (lower) of **(A)** total potassium (I_peak_), **(B)** transient outward (I_to_), and **(C)** ultra-rapid (I_Kur_) currents. **(D)** I–V curves of K1 and steady state currents (I_K1_/I_Kss_, WT black and TG red lines, WT *n* = 8 and TG *n* = 17; from four animals). Refer to insets for voltage-step protocols (upper without and lower with inactivation prepulse). **(E)** Gene expressions of potassium channel subunits (WT *n* = 7 and TG *n* = 6). ^*^P < 0.05, ¤*P* < 0.01 and #*P* < 0.001.

In general, the AP repolarization is determined by the potassium currents, of which transient outward and delayed rectifier currents are the most prominent ones in mouse ventricular myocytes (Nerbonne, 2004). While there was relatively small change in the total K^+^ current (~18%; Figure 6A), VEGF-B overexpression shown drastic decreasing of transient outward current (I_to_, Figure 6B). Moreover, decreasing of I_to_ in VEGF-B animals was accompanied with significant acceleration of inactivation of this current (by ~1.7-fold). The ultra-rapid component of delayed rectifier current as well as I_K1_ and I_Kss_ were unaffected (I_Kur_, I_K1_, and I_Kss_ Figures 6C,D). Channels responsible for fast and slow I_to_ are assembled by α-subunits K_v_4.2 and K_v_1.4, encoded by genes *Kcna4* and *Kcnd2*, respectively (Nerbonne, 2004). However, expression of these genes was not different between WT and TG myocytes; instead, we saw downregulation of *Kcnip2* K_v_ channel-interacting protein 2 (ß-subunit), a known modulatory subunit of the K_v_4.2/3 channel (Figure 6E). This indicates that VEGF-B regulates AP repolarization partly by affecting the expression of potassium channel subunits and accessory proteins.

### Effects of VEGF-B-related alterations on electrophysiology

**Figure 7:**
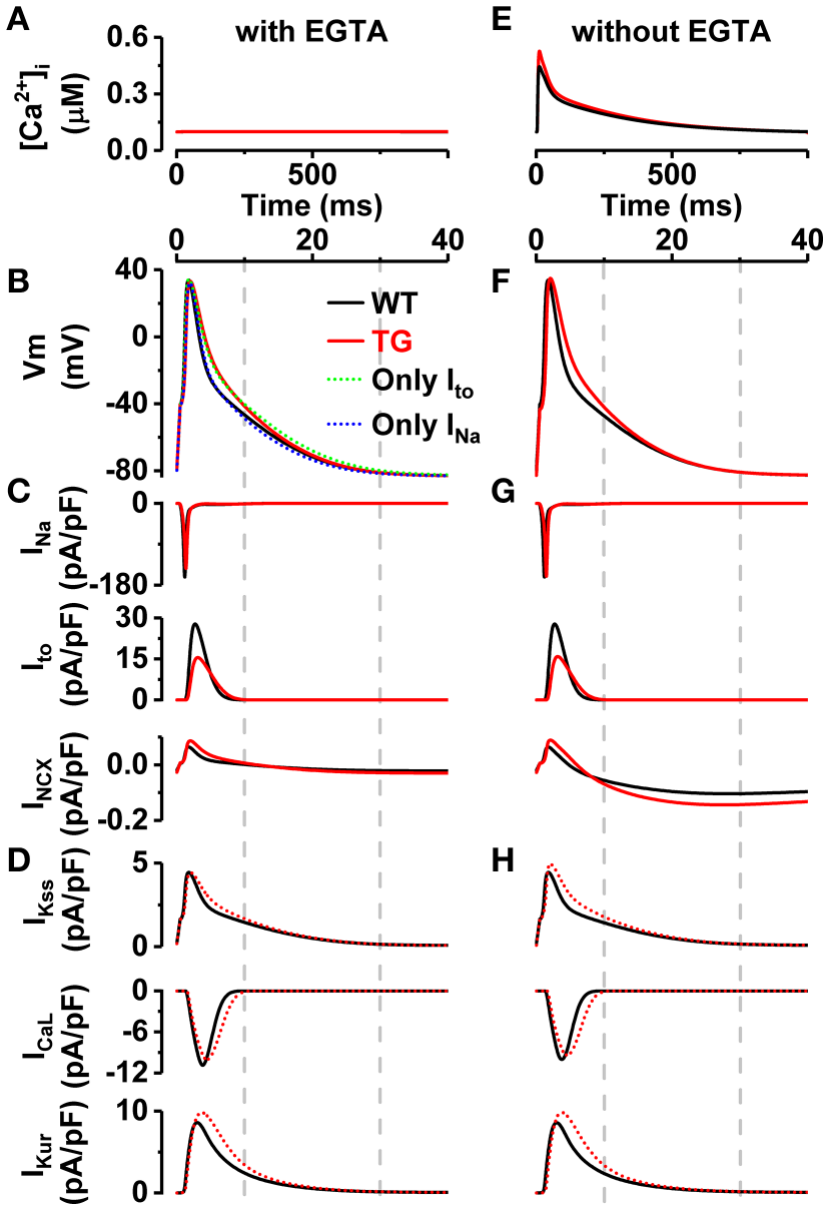
Effect of VEGF-B-related electrophysiological alterations explored *in silico*. From top to the bottom: **(A,E)** [Ca^2+^]_i_ transients, **(B,F)** action potentials, **(C,G)** currents introduced into model I_Na_, I_to_, I_NCX_ (solid red lines), and **(D,H)** resulting currents I_Kss_, I_CaL_, I_Kur_ (dotted red lines) in simulated pacing experiments, in the presence **(A–D)** and absence **(E–H)** of EGTA. Note, the different time scale in [Ca^2+^]_i_ transients panels. The VEGF-B TG model variant (red lines) accounts for the (1) decreased I_Na_ (−18%), (2) reduced I_to_ (conductance −49%, and inactivation rate +59%), and (3) increased NCX current (+36%) as found in experiments.

To elucidate the isolated contribution of VEGF-B-related modifications of I_Na_, I_to_, and I_NCX_ on electrophysiology (Figures 5, 6), we replicated the AP experiments (Figure 4) *in silico*, together with the effect of EGTA Ca^2+^ buffer used in experiments (Figure 7A), using a computational model of mouse ventricular myocyte (Koivumaki et al., 2009). Model simulations recapitulated well the emergent changes in the AP morphology (Figure 7B). As the comparison of simulated APs shows (Figure 7B), the sodium and potassium channel modifications partially cancel out each other, and thus the sum effect on repolarization is smaller in the virtual VEGF-B myocyte. The reduced I_Na_ (Figure 7C) lead to a 12.0% decrease in maximum upstroke velocity of the AP. The indirect effect of slower AP repolarization is that the inactivation of I_Kss_, I_CaL_, and I_Kur_ is decelerated (Figure 7D).

Furthermore, we studied the possible interdependence of AP changes and the apparent changes in CaT amplitude with simulations of more physiological conditions (without EGTA; Figure 7E). While the enhanced NCX activity (Figure 7G) should result in increased Ca^2+^ extrusion and thus reduced CaT amplitude, the simulations demonstrate that due the reduced I_to_ the initial repolarization phase is much slower (Figure 7F), which decelerates the inactivation of I_CaL_ (Figure 7H) and thus increases the integral of Ca^2+^ influx by +39%. As a result, the CaT amplitude is increased by 24% (Figure 7E).

## Discussion

Here, we studied the effect of VEGF-B overexpression in mouse heart at the level of cardiac myocytes. We report that cardiac-specific overexpression of VEGF-B induces mild changes in cardiomyocyte calcium signaling and growth without pathological metabolic remodeling. Interestingly, our results show that VEGF-B induces marked changes in the electrophysiological properties of cardiomyocytes, including changes in densities of both depolarizing and repolarizing currents, ECG, and action potential parameters.

Particular changes in mouse ECG complex correlate with certain cardiac conditions such as aging, infarction, and LV hypertrophy (LVH) (Merentie et al., 2015); these changes can be used to estimate disease progression (Sysa-Shah et al., 2015). VEGF-B TG hearts show two distinct ECG changes. First is increase in the width of the QRSp which together with unaffected QRS represents prolongation of early repolarization (Liu et al., 2004; Boukens et al., 2013, 2014). That prolongation fits very good to lengthening of APD_60–70_ which we observed in TG cardiomyocytes. That change of QRSp is common to LVH (Merentie et al., 2015) and some forms of sodium channelopathies (Remme et al., 2006; Watanabe et al., 2011). And second is decreased amplitude of the R and S waves, which has been shown to associate with acute infarction or ischemia in mouse heart (Gehrmann et al., 2001; Merentie et al., 2015) and e.g., sodium channel blocker Ajmaline (Boukens et al., 2013). VEGF-B TG hearts show only modest signs of organ level hypertrophy and no infarctions, so that the ECG anomalies cannot be considered as signs of cardiac disease, but they rather reflect more specific changes in cardiomyocytes electrophysiology. Interpretation of mouse ECG is not straightforward (Boukens et al., 2014), but ECG changes of the VEGF-B mice shown here suggest changes in both depolarizing and repolarizing currents, which predispose the heart to arrhythmias upon stress. Cell level electrophysiological changes such as AP prolongation predispose cardiomyocytes to afterdepolarization and triggered arrhythmias (Janse, 2004; Aiba and Tomaselli, 2010). Consequently, APD changes are common findings in heart diseases, such as heart failure and hypertrophy (Wickenden et al., 1998), and in a variety genetic mouse models of human long QT syndrome (Salama and London, 2007). Due to short APD in mouse ventricular cardiomyocytes, the depolarizing currents such as L-type Ca^2+^ current contribute less to APD than in species with long AP, such as humans. Instead, mouse AP repolarization is dominated by potassium currents, especially transient outward currents (I_to,f_, I_to,s_) (Nerbonne, 2004). VEGF-B TG myocytes have an increase in APD due to decreased I_to_. At the level of K^+^ channel transcripts, TG myocytes have suppressed expression of *Kcnip2*. Although translation of ion channel transcripts to functional channels is subject to various post-translational modifications and multiple regulative steps in channels assembly, the protein resulting from *Kcnip2* transcript has been shown to be a regulator of I_to_ (Kuo et al., 2001) which is in line with our findings and corresponds well with the previous findings from *Kcnip2*^−/−^ mice (Thomsen et al., 2009).

Some of the ECG and action potential findings from VEGF-B TG hearts cannot be solely explained by the changes in potassium currents. These include a decrease in the amplitude of the ECG complex (R and S waves), increased duration of the QRSp, and the absence of QT lengthening usually associated with an increase in APD. These changes suggested that the depolarization phase of the ventricles of VEGF-B mice is also affected and the ECG changes reflect combinatory effects of both depolarizing and repolarizing currents of the cardiomyocytes. In line with this, action potentials of VEGF-B mouse ventricular myocytes not only had increased duration, but also a decrease in maximum upstroke velocity as well as an increase in the rise time of the initial depolarization. The upstroke phase of the AP is dependent on the Na^+^-current density and therefore it was not a surprise that it was accompanied by a reduction in I_Na_ density and a slight rightward shift in the activation-inactivation curve of the I_Na_ in TG cardiomyocytes. Unlike the potassium channel remodeling, I_Na_ changes are not commonly associated with cardiac hypertrophy or cardiac failure progression (Hill, 2003). Interestingly, according to previous data from mouse models where the I_Na_ downregulation occur as a secondary effect (Gavillet et al., 2006; Hesse et al., 2007; Cerrone et al., 2012; Rizzo et al., 2012; Han et al., 2015), or as a result of expression of mutated Na_v_1.5 (Remme et al., 2006; Abriel, 2007; Watanabe et al., 2011), the electrophysiological findings in mouse heart are similar to those in VEGF-B TG mice. Prototypically these include decreases in the QRS complex amplitude, reduced upstroke velocity of the AP, and reduced conduction velocity. Therefore, it appears that the electrophysiological phenotype of the VEGF-B TG hearts is greatly impacted by the downregulation of the Na^+^-current. According to the simulations with a mathematical model, the measured changes in I_to_ and I_Na_ densities together with the changes in their activation/inactivation properties explain well the measured changes in the AP shape. Interestingly, the modeling also suggests that the modest changes in calcium transient amplitude at low pacing frequencies actually results from the AP lengthening. That is, slower initial AP repolarization decelerate the inactivation of the I_CaL_ and consequently increases to total Ca^2+^ influx via I_CaL_ during AP in VEGF-B TG myocytes.

The effects of VEGF-B on the structure and function of the heart have been studied with a wide variety of animal models and approaches (Bry et al., 2014). It has become evident that some of the VEGF-B effects show species or isoform specificity, whereas others depend on methods used for overexpression or knockdown. The TG mice analyzed in this study showed a relatively mild tendency to hypertrophy, indicating that the effect of VEGF-B on cardiac phenotype is graded, producing milder changes with less powerful transgene expression. Collectively the existing data show that VEGF-B induces a spectrum of changes in the heart ranging from capillary density and structure and growth of the myocardium to more diffuse changes in energy substrate metabolism and signaling. Bearing this in mind it is a surprise that the cardiomyocyte-level phenotype of the VEGF-B TG mice show minimal alterations in Ca^2+^ signaling, contraction, or structure. Instead, TG hearts have a very distinct and specific electrophysiological phenotype originating from precise changes in ion current densities. This suggests that VEGF-B triggers pathways mediating cardiomyocyte-specific alterations in electrophysiology without directly affecting the contractile function of the whole heart.

## Author contributions

PT, JH, and NN designed the research. SY, KA, and RK provided the material. NN, JH, TT, MM, JK, and EG conducted the experiments, collected, and analyzed data. PT and NN wrote the manuscript. All authors read and approved the final version of the manuscript.

### Conflict of interest statement

The authors declare that the research was conducted in the absence of any commercial or financial relationships that could be construed as a potential conflict of interest.
