# Supplementary material for: Vascular Endothelial Growth Factor-B Induces a Distinct Electrophysiological Phenotype in Mouse Heart
Source: Front Physiol. 2017 May 31;8:373. doi: 10.3389/fphys.2017.00373 (PMC5450225; doi:10.3389/fphys.2017.00373)
Supplement: Supplementary file 1 [file DataSheet1.DOCX]

Supplementary Material

**VASCULAR ENDOTHELIAL GROWTH FACTOR-B INDUCES A DISTINCT ELECTROPHYSIOLOGICAL PHENOTYPE IN MOUSE HEART**

Nikolay Naumenko, Jenni Huusko, Tomi Tuomainen, Jussi T. Koivumäki, Mari Merentie, Erika Gurzeler, Kari Alitalo, Riikka Kivelä, Seppo Ylä-Herttuala, Pasi Tavi^*^

* Correspondence: Pasi Tavi [pasi.tavi@uef.fi](mailto:pasi.tavi@uef.fi)

# Supplementary Methods

- 1. **Experimental animals**

Two to four months old transgenic mice overexpressing human VEGF-B167 and VEGF-B186 isoforms (Fig. S1) under the cardiac-specific myosin heavy chain (MHC) promoter (Bry et al. 2010) were used. MHC-GenB mice were bred into the C57Bl/6JOlaHsd background for over 10 generations. TG-negative littermates were used as controls in all experiments. The animals were kept in standard housing conditions in the National Laboratory Animal Centre of The University of Eastern Finland. Diet and water were provided *ad libitum*. All animal experiments were performed according the guidelines of Directive 2010/63/EU of the European Parliament on the protection of animals used for scientific purposes, the procedures were also approved by the Animal Experiment Board in Finland and carried out in accordance with the guidelines of the Experimental Animal Committee of the University of Eastern Finland.

**1.2. Echocardiography and electrocardiography**

Ten MHC-GenB and ten TG-negative littermate controls were used for echocardiographic and electrocardiographic measurements performed with the Vevo2100 Ultrasound System (VisualSonics Inc., Toronto, ON, Canada). A high-frequency ultrasound probe (MS400) operating at 30 MHz with a minimum of 300 frames per second was used. The animals were anaesthetized with isoflurane (induction: 4.5 % isoflurane, 450 mL air, maintenance: 2.0 % isoflurane, 200 mL air, Baxter International Inc., Deerfield, IL, USA). Mice were placed in a supine position on a heated platform (THM100, Indus Instruments, Houston, TX, USA) to maintain the body temperature at 36-37 ºC. Ejection fraction (EF), left ventricle anterior wall thickness (LVAW), left ventricle volume (LV Vol) and left ventricle mass (LV Mass) were determined from parasternal short axis M-Mode measurements. EF was calculated by Vevo2100 software using the Teicholz formula.

To obtain the ECG signal, the paws of the mice were connected to the electrode pads on the platform using ECG gel and fixed with a skin tape. The recorded ECG represents the standard limb lead II. The mouse heart rate and respiration were monitored during anaesthesia via ECG pads. The raw data of ECG were analysed with a Matlab-based ECG analysis program (Kubios HRV, version 2.0 beta 4, Department of Physics, University of Eastern Finland, Kuopio, Finland), which was modified specially for analysing mouse ECG (Merentie et al. 2015). Time intervals (QRS, QRSp and QTc time) and amplitudes of R and S wave were analysed from the mean curve generated from a 30-second ECG recording. QTc time was calculated as QT ms / (R-R_0_ ms /100 ms)^½^  (Mitchell et al. 1998).

**1.3. Langendorff isolated heart perfusion model**

All experiments were performed on the setup produced by Hugo Sachs Elektronik (Germany). Mice were killed by cervical dislocation and their hearts were rapidly excised. Then hearts were perfused via aorta in the Langendorff mode at a constant flow of 1.6 - 2 mL/min. The composition of Krebs-Henseleit (KH) buffer for the perfusion (37^o^C) was (in mM): NaCl 118, KCl 4.7, KH_2_PO_4_ 1.2, MgSO_4_ 1.2, CaCl_2_ 3, NaHCO_3_ 25, glucose 11, and it was gassed continuously with a mixture of 95% O_2_ and 5% CO_2_ (pH 7.4). Pacing by surface electrodes with a frequency 8 Hz was used in all experiments. Left ventricular pressure (LVP) was measured by a PVC balloon catheter inserted into the left ventricle. Heart rate and LVP were monitored with an HSE ISOTECH pressure transducer and acquired to the PC using the data acquisition board (National Instruments, Austin, TX, USA) and WinEDR free software (Strathclyde University, UK). Initial LVP (diastolic pressure) was adjusted by balloon volume at 5-7 mmHg. Left ventricular developed pressure was calculated as the difference between systolic and diastolic pressures of the LVP trace. Experiments investigating palmitate consumption by the heart were performed. The bovine serum albumin (BSA)-based KH buffer was used in these experiments (Belke et al. 1999). The fatty acid was dissolved in a mixture of water, ethanol and sodium carbonate and was boiled. The palmitate solution left after evaporation of ethanol was mixed with a warm (but not boiled) solution of 3% BSA, to bind palmitate. The final concentration of palmitate was 1.2 mM. This solution was prepared by dissolving BSA in KH buffer without glucose one day before the experiment. The glucose was added to the solution right before the experiment. The heart perfusion was started with ordinary KH buffer, which later was changed to palmitate-containing buffer without glucose.

**1.4. Single cell isolation**

Adult mouse ventricular myocytes were obtained by enzymatic dissociation as described before (AfCS Procedure Protocol PP00000125). Briefly, isolated hearts were placed in a Langendorff apparatus for perfusion (37°C, 3 mL/min) with a trypsin (Sigma) and liberase (Roche Applied Science) solution. After that, ventricles were cut into small pieces and gently minced with a Pasteur pipette. The concentration of Ca^2+^ in solution was increased slowly up to 1 mM. The suspension of cells was placed on laminin-coated coverslips and stored in an incubator (5% CO_2_ at 37°C).

**1.5. Size measurement of the isolated cardiomyocytes**

Adult cardiomyocyte size was assessed with a Coulter Counter Z2 (Beckman Coulter) equipped with a 200 µm aperture and data analyzed with Accucomp software (Beckman Coulter). 20% of the isolated cells from one heart were suspended in 2 mL medium and 10 mL Beckman Isoton II diluent (Beckman Coulter) was added. The metered volume was 1 mL, main gain 32 and aperture current 0.250. From each analysis mean and median cell volumes of the cell population above the size of 20000 fL were recorded. The cell population below 20000 fL (equals to a spherical particle with 17 µm diameter) was contaminated with smaller cells and cell debris, and therefore discarded from the analysis (see Fig. S2).

**1.6. Western blot**

Frozen ventricular tissue was lysed with a TissueLyser II (Qiagen) in buffer A (20 mM Tris-HCl (pH 7.5), 10 mM NaCl, 0.1 mM ethylenediaminetetracetic acid (EDTA), 0.1 mM ethylene glycol-bis(β-amino ethylether) tetra-acetic acid (EGTA), 1 mM β-glycerophosphate, 1 mM Na_3_VO_4_, 2 mM benzamidine, 1 mM phenylmethylsulfonyl fluoride, 50 mM NaF, 1 mM dithiothreitol, 10 µg/mL leupeptin, 10 µg/mL pepstatin, 10 µg/mL aprotinin). To remove remaining tissue debris, the solution was centrifuged 1 min at 400 g and supernatant collected for further processing.

For total protein isolation, one part of buffer B (100 mM Tris-HCl (pH 7.5), 750 mM NaCl, 5 mM EDTA, 5 mM EGTA, 5% Triton-X100, 12.5 mM sodium pyrophosphate, 5 mM β-glycerophosphate, 5 mM Na_3_VO_4_) was added to four parts of tissue lysate. After thorough mixing with a vortex mixer, the sample was centrifuged 20 min at 15000 g and supernatant was collected as a total protein sample. For separation of cytoplasmic and membrane protein fractions, tissue lysate in buffer A was first centrifuged 20 min at 15000 g. Supernatant was collected as a cytoplasmic protein fraction. Pelleted material was dissolved in 0.2X buffer B and sonicated three times for 30 s. After sonication, the solution was centrifuged 20 min at 15000 g and supernatant collected as a membrane protein fraction. Protein concentration was measured with the Bio-Rad Protein Assay.

One part of sodium dodecyl sulfate (SDS) buffer (315 mM Tris-HCl (pH 6.8), 50% glycerol, 2% SDS, 1.25 M dithiothreitol, 0.05% bromophenol blue) was added to four parts protein sample and the solution was incubated 4 min at 95°C. 10-50 µg of protein was loaded onto an 8-12% SDS polyacrylamide gel. After gel electrophoresis, proteins were transferred to a nitrocellulose membrane (0.2 µm, Bio-Rad Laboratories), which was blocked with 5% BSA. For blotting of the large (240 kDa) Na_v_1.5 protein, a gradient gel (4-15%, Bio-Rad Laboratories, #456-1083) was used and protein samples transferred to a 0.4 µm membrane. Membranes were incubated with primary antibody followed by incubation with Cy5 (GE Healthcare, PA45012) or Cy3 (PA43010)-linked secondary antibody. Blots were visualized with the ECL Plus Detection Kit (GE Healthcare) using a Typhoon 9400 scanner (GE Healthcare). Antibodies used were: anti-Na_v_1.5 (Alomone labs, ASC-005), anti-VEGF-B (R&D Systems, Af751) and β-actin (Cell Signaling Technology, 4967). Ponceau S staining solution (Sigma-Aldrich, St. Louis, MO, USA) was used as per manufacturer's instructions to visualize total membrane protein as a loading control for the blotted Na_v_1.5 protein.

**1.7. Metabolic analysis of isolated cardiomyocytes with a Seahorse extracellular flux analyzer**

Freshly isolated cardiomyocytes were plated on XF24 Cell Culture Microplates (Seahorse Bioscience) in 500 µl of XF Assay Medium (Seahorse Bioscience) (pH 7.4) containing 10 mM 2,3 Butanedione monoxime (BDM). Plates were coated with 3 µl Matrigel (BD Matrigel matrix, growth factor reduced, BD Biosciences) diluted 1:1 in XF Assay medium. For cell attachment, cells were incubated at 37°C in a humidified CO_2_-free incubator for 1 hour before the analysis with a Seahorse XF24 Analyzer (Seahorse Bioscience). Assay buffer was supplemented with 200 μM sodium palmitate (Sigma, P9767) conjugated to bovine serum albumin (BSA) (fatty acid free, Sigma, A8806), or with 4.5 g/L glucose and unconjugated BSA. 1.2 mM palmitate conjugated to BSA was prepared in Krebs-Henseleit buffer as described earlier (Belke et al. 1999) and diluted 1:6 in XF assay medium to achieve a solution with 200 μM palmitate. After calibration of the assay cartridge, the cell plate was placed in the analyzer and three consecutive measurements of basal respiration were performed with the following cycle of commands: 3 min mixing, 2 min waiting and 3 min measuring. To assess the maximal mitochondrial respiration, 1.5 µM carbonyl cyanide-4-(trifluoromethoxy)phenylhydrazone (FCCP) was added to the assay medium. Addition of FCCP solution via injection ports of the assay cartridge during the assay was noticed to lead to detachment of the cells. In order to avoid this, the assay was stopped and FCCP solution carefully pipetted into the wells. A second assay for maximal respiration was performed by using the same assay cartridge and calibration solution as in the first assay. Respiration of the cells after FCCP addition was measured ten times with the following cycle: 4 min mixing and 1.5 min measuring. Results were normalized to total protein of the analyzed cells.

**1.8. Gene expression.**

Primers and probes designed to detect mouse mRNA transcripts in Taqman based RT-qPCR are listed in Table S1.

**1.9. Patch-clamp experiments**

All experiments were carried out at 37°C (20°C for sodium current experiments) (TC2BIP, Cell MicroControls, USA). Only quiescent elongated rod-shaped cells with cross-striations were used for recordings. Coverslips with attached cells were transferred to the recording chamber (Cell MicroControls, USA, flow rate approx. 1-2 mL/min, chamber volume 0.4 mL) perfused with Dulbecco’s modified Eagle medium plus glutamax I (Invitrogen, DMEM) or Tyrode solution containing (in mM): 130 NaCl, 5.4 KCl, 1 CaCl_2_, 1 MgCl_2_, 0.3 Na_2_HPO_4_, 10 HEPES, and 5.5 glucose, pH 7.4 with NaOH. After establishment of whole-cell access the medium was switched to recording solution. HEPES-buffered solutions were bubbled with 100% O_2_. Carbogen gas (95% O_2_, 5% CO_2_) was used for DMEM. Whole-cell voltage-clamp (Axopatch 200B, Digidata 1440A, Molecular Devices Inc., USA) was used for currents and current-clamp (I=0) for action potential (AP) recordings. Patch electrodes (Harvard Apparatus, United Kingdom) were pulled and fire polished with Sutter P-97 (Sutter Instrument Company, Novato, CA). Patch electrodes had resistances from 1.5 to 3 MΩ when filled with pipette solution. Recordings were carried out after a membrane rupture of 5 min. The cell capacitance and series resistance were compensated electronically. The cells with an unstable or high access resistance (larger than 10 MΩ) were discarded. Typically, the access resistance was 3-6 MΩ. Under voltage clamp control cells were held at -80 mV (-120 mV for sodium current experiments). Membrane capacitance and resistance were estimated in response to a 5 mV pulse. The current amplitudes were normalized by cell capacitance. Recordings were carried out at a sampling rate of 10 kHz (20 kHz for sodium current recordings) and a low-pass Bessel filter at 5 kHz.

**AP recordings.** APs were elicited (1-ms current injection) and recorded using the current-clamp mode. The stimulus frequency was 0.5 Hz. The intracellular solution contained (Yang et al. 2005) (in mM): 120 K-aspartate, 25 KCl, 1 MgCl_2_, 2 Na_2_-phosphocreatine, 4 Na_2_-ATP, 2 NaGTP, 10 EGTA, and 5 HEPES (pH 7.2 with KOH) and the bath solution was DMEM. AP parameters were analyzed from AP recordings that reached the steady state shape and amplitude.

**L-type Ca-current recordings.** To characterize the L-type Ca^2+^ current we used the protocol described previously (Xu et al. 2011). The internal solution contained (in mM): 110 CsOH, 90 aspartic acid, 20 CsCl, 10 tetraethyl ammonium chloride (TEA chloride), 10 HEPES, 10 EGTA, 5 Mg-ATP_2_, 5 Na_2_-creatine phosphate, 0.4 GTP-Tris, 0.1 leupeptin (pH 7.2 with CsOH) and the bath solution contained (in mM): 125 *N*-methyl-glucamine, 5 4-aminopyridine (4-AP), 20 TEA chloride, 2 CaCl_2_, 2 MgCl_2_, 10 glucose and 10 HEPES (pH 7.4 with HCl). After an initial 1-sec prepulse at -40 mV, Ca^2+^ currents were elicited using 200-ms voltage steps from -30 to +50 mV in 10-mV increments. Voltage-dependence of inactivation was assessed by holding cells at various potentials from -40 to +10 mV for 2 sec followed by a 100-ms test pulse to +10 mV.

**K^+^ currents recordings.** The protocol for potassium currents measurement was adopted from Rivard et al. (2009). The internal solution contained (in mM): 110 K-aspartate, 20 KCl, 8 NaCl, 1 MgC1_2_, 1 CaC1_2_, 10 BAPTA, 4 K_2_ATP and 10 HEPES (pH 7.2 with KOH). The bath solution was Tyrode (see above). The total K^+^ current (I_peak_) was elicited by a series of 500-ms voltage steps varying from -110 to +50 mV in 10-mV increments at a frequency rate of 0.2 Hz. The current density of the inward rectifier K^+^ current (I_K1_) was determined at the end of the voltage steps, ranging from -110 to -40 mV. To eliminate the transient outward K^+^ current (I_to_) an inactivating voltage prepulse was applied (50 ms, -40 mV). The remaining current consisted of the ultra-rapid delayed rectifier K^+^ current (I_Kur_) and the steady-state outward K^+^ current (I_Kss_). We then applied 100 µM 4-AP (which blocks I_Kur_) to record I_Kss_. The density of I_to_ was obtained by off-line subtracting the current traces measured with and without the inactivating prepulse, while I_Kur_ was measured as a subtraction of currents recorded in the absence and presence of 4-AP. The current densities of each of the components of the outward K^+^ currents were determined at the peak current, excluding I_Kss_ which was evaluated at the end of test pulse.

**Na^+^ current.** The solutions and protocol for the I_Na_ recordings were as described previously (Sato et al. 2009). The recordings were carried out at 20°C (TC2BIP, Cell MicroControls, USA). The internal solution contained (in mM): 5 NaCl, 135 CsF, 10 EGTA, 5 Mg-ATP, 5 HEPES, pH 7.2 with CsOH and the bath solution contained (in mM): 20 NaCl, 117.5 CsCl, 1 CaCl_2_, 1 MgCl_2_, 0.1 CdCl_2_, 11 glucose, 20 HEPES, pH 7.4 with CsOH. Series resistance was compensated electronically down to 1 MΩ. To characterize the voltage-dependence of the peak I_Na_, single cells were held at -120mV and 200 ms voltage steps were applied from -80 to +40mV in 5 mV increments with 3-sec inter sweep intervals. The voltage-dependence of inactivation was assessed by application of 500-ms prepulses of different voltage amplitudes (in the range from -110 mV to -40 mV), which were followed by 30-ms test pulse to -40 mV. A standard two-pulse protocol was studied to evaluate recovery rate from inactivation. Two 20-ms pulses to -40mv separated by different time increments from 1 to 90 ms were applied.

**Late Na^+^ current.** Late I_Na_ was estimated as integration of the current from 50 to 250 ms of the beginning of the depolarizing pulse (250 ms) without and with ranolazine (10 µM) in the bath solution. In some experiments to estimate late I_Na_ we used bath solution contained physiological concentration of Na^+^ (Sossalla et al. 2010), the voltage step was -40 mV from holding potential of -120 mV.

Current-voltage relations for steady-state activation and inactivation for calcium and sodium currents were determined by fitting a Boltzmann function (*I/Imax =* [1+exp ((*V-V½*)/*k*)]^–1^), yielding the membrane potential of the half-maximal activation (*V½-*activation) and inactivation (*V½*-inactivation) and slope factor (*k*). The current recovery from inactivation curves were fitted using a single exponential function. Data analysis was made using Clampfit10 software (Molecular Devices Inc., USA).

**1.10. Intracellular Ca^2+^ measurements**

Cardiomyocytes were loaded with Fluo-4-acetoxymethyl (AM)-ester (10 μM, 0.02% pluronic F-127, Invitrogen) in DMEM for 30 min in an incubator (37°C, 5% CO_2_) and then coverslips with attached cells were placed into the recording chamber (see above). Experiments were carried out after a period of 20 min to allow deesterification of the dye. [Ca^2+^]_i_ measurement was performed with a confocal inverted microscope (FluoView 1000; Olympus, Japan). To measure myocyte calcium signals ([Ca^2+^]_i_ - transients) the cells were excited at 488 nm and the emitted light (500-600 nm) was collected through water immersion 40X or 60X objective lens and line-scan mode was used. To stimulate the cells, myocytes were paced with 1-ms voltage square pulses (Grass stimulator, S48) 50% over the excitation threshold through two platinum wires located on both sides of the chamber. Stimulus frequency was within the range of 0.5 Hz to 10 Hz. In some experiments caffeine (10 mM, Sigma) was applied directly to the studied area by a local perfusion manifold (Cell MicroControls, USA). Fluo-4 fluorescence intensity is expressed as an F/F_0_ - ratio, where F is the background subtracted fluorescence intensity and F_0_ is the background subtracted minimum fluorescence value measured from each cell at rest. The images were analyzed with FluoView and ImageJ (imagej.nih.gov/ij/) software.

**1.11. Mathematical model and simulations**

We have used our previously published *in silico* model of mouse ventricular myocyte to evaluate the contribution of individual VEGF-B related changes on AP morphology (Koivumaki et al. 2009). The VEGF-B related mutant model variants were implemented by modifying the parameters of 1) fast sodium current, 2) transient outward potassium currents, and 3) sodium-calcium exchanger according to experimental findings. I_Na_ conductance was reduced 0.89-fold, steady-state activation/inactivation curves were shifted to the right by 2.76/1.38 mV. I_to_ conductance was reduced 0.506-fold, and inactivation rate was increased by 1.59-fold. Maximum NCX current was increased 1.36-fold. The AP data was obtained for each model variant at a steady-state, after 5 minutes of pacing. The extra- and intracellular ion concentrations were adjusted to match the *in vitro* measurement protocols, including Ca^2+^ buffering with EGTA.

**1.12. Data and statistical analysis**

Data and statistical analyses were made using Origin 9 software (OriginLab Corp., Northampton, MA, USA). For statistical analysis, one-way ANOVA with Fisher’s post-hoc comparison was performed for all currents and calcium transient frequency dependence, as well as Student’s t tests where appropriate, were applied at a level of significance of P<0.05. Data are given as mean values ± SEM.

# Supplementary Figures and Tables


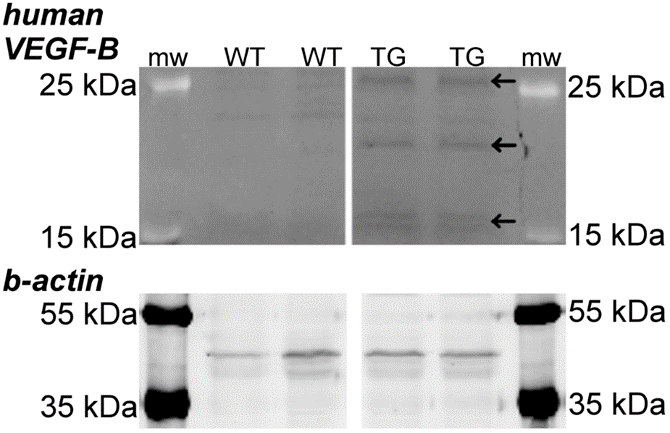


**Supplementary Figure S1.** Human VEFG-B protein is expressed in transgenic animals. Western blot membrane stained with antibody specific to human VEGF-B (Af751, R&D Systems) shows the expression of 18, 23 and 28 kDa VEGF-B proteins (arrows) in transgenic but not in wild type animals. B-actin stained from the same membrane.





**Supplementary Figure S2. Cell size measurement.** Representative histograms from the analysis of cell size with a Coulter Counter. Below the size of 20 pL the cell population is contaminated with smaller cells and cell debris. Patterned square points out the area of the plot that is shown in Fig.1B and the cell population that was used in the analysis.

**Supplementary Figure S3. Expression of connexins and Ca^2+^ handling genes in WT and VEGF-B TG hearts.** **(A)** Expressions of connexins *Gja1, Gja5, Gjc1 and Gjd3* (*Cx43, Cx40, Cx45* and *Cx30.2*, respectively) and **(B)** left panel: [Ca^2+^]_i_ – handling related genes: sodium/calcium exchanger (*Slc8a1*), SR calcium ATPase (*Atp2a2*), phospholamban (*Pln*) and ryanodine receptor (*Ryr2*) and the *Atp2a2/Pln* - ratio in ventricular cardiomyocytes (right panel). * *P*<0.05.

**Supplementary Figure S4. Late Na^+^ - current in ventricular cardiomyocytes.** Left panel: Late I_Na_ before and after ranolazine application. Refer to inset for voltage-step protocols. Right panel: enlarged average superimposed sodium currents obtained at physiological [Na^+^]_out_ (grey area is SEM, WT n=17 and TG n=26).

**Supplementary Table S1. Sequences of the primers and probes used in Taqman-based real-time quantitative PCR.**

| **Short name, (AKA)** | **Name, Gene ID** | **nucleotide sequence** | |
| --- | --- | --- | --- |
| *Acta1*  *(SkA)* | actin, alpha 1, skeletal muscle | 5' primer  3' primer | TCCTCCGCCGTTGGCT  AATCTATGTACACGTCAAAAA |
|  | 11459 | fluorogenic probe | CATCGCCGCCACTGCAGCC |
| *Atp2a2*  *(Serca2a)* | ATPase, Ca++ transporting, cardiac muscle, slow twitch 2 | 5'  3' | CAGCCATGGAGAACGCTCA  TCGTTGACCCCGAAGTGG |
|  | 11938 | probe | ACAAAGACCGTGGAGGAGGTGCTGG |
| *Cacna1c* | calcium channel, voltage-dependent, L type, alpha 1C subunit 12288 | 5'  3'  probe | TTGACAATGTTTTGGCAGCC  TCTGGCCACCCTTCGA  TGATGGCTCTCTTCACCGTCTCCACC |
| *Cpt1b* | carnitine palmitoyltransferase 1b, muscle | 5'  3' | GACAGAAGCAAACCTGAG  GTCACAGTGAACTGGAAA |
|  | 12895 | probe | CTACTGCCTGGTGTGCTTCC |
| *Gja1* | gap junction protein, alpha 1 | 5' | AAGGGAAGAAGCGATCCTTACC |
| *(Cx43)* | 14609 | 3' | CGCAGTCTTTGGATGGGCT |
|  |  | probe | CGCCACCACCGGCCCACT |
| *Gja5* | gap junction protein, alpha 5 | 5' | GGCACCCTACTCAACACCTATGT |
| *(Cx40)* | 14613 | 3' | CGATGAAGGCCACCTCCAT |
|  |  | probe | TGCACCATTCTGATCCGCACCAC |
| *Gjc1*  *(Cx45)* | gap junction protein, gamma 1 | 5'  3' | ACAAGCAAAACAAAGCCAATATTG  CAGCCGGGAGGTGTTCCT |
|  | 14615 | probe | CCAGGAACAGCAGTACGGCAGCCA |
| *Gjd3* | gap junction protein, delta 3 | 5' | CGCACACGGTCGACTGTTT |
| *(Cx30.2)* | 353155 | 3' | GGCGAAGTAGAAGACCACGAA |
|  |  | probe | AGCCGGCCCACCGAGAAGACC |
| *Kcna4* | potassium voltage-gated channel, shaker-related subfamily, member 4 16492 | 5'  3'  probe | GTGGAAAAGGGGAAACAA  CAGGAAATGAAGAGCATCC  ATTCACAAGAAGCACTTCACCATTCC |
| *Kcna5* | potassium voltage-gated channel, shaker-related subfamily, member 5 16493 | 5'  3'  probe | GAGTCATCCTCTTCTCCA  TAGCCTACAGTGGTCATA  TACTTCGCAGAGGCAGACAATCA |
| *Kcnd2* | potassium voltage-gated channel, Shal-related family, member 2 16508 | 5'  3'  probe | CCTTCTGGTACACCATCG  GACTGAAGTTCGACACGA  CGCAATGACCAAGACTCCGC |
| *Kcnip2* | Kv channel-interacting protein 2 | 5'  3' | CAGCAACTATGCTACTTTTCTCTTCAA  AGCCACAAAGTCCTCAAAACTGA |
|  | 80906 | probe | CCTTTGACACCAACCACGATGGCTC |
| *Myh6*  *(aMhc)* | myosin, heavy polypeptide 6, cardiac muscle, alpha | 5'  3' | GGTGCCAAGAAGATGCACG  TTATGTTTATTGTGTATTGGCCACAG |
|  | 17888 | probe | CGAGGAATAACCTCTCCAGCAGACCCTC |
| *Myh7*  *(bMhc)* | myosin, heavy polypeptide 7, cardiac muscle, beta | 5'  3' | AGCTCTAAGGGTGCCCGTG  TGCTTCCACCTAAAGGGCTG |
|  | 140781 | probe | AGCCCTCAGACCTGGAGCCTTTGC |
| *Nppa* | natriuretic peptide type A | 5' | GAAAAGCAAACTGAGGGCTCTG |
| *(Anp)* | 230899 | 3' | CCTACCCCCGAAGCAGCT |
|  |  | probe | TCGCTGGCCCTCGGAGCCT |
| *Nppb* | natriuretic peptide type B | 5' | AGGCGAGACAAGGGAGAACA |
| *(Bnp)* | 18158 | 3' | GGAGATCCATGCCGCAGA |
|  |  | probe | CATCATTGCCTGGCCCATCGC |
| *Pfkm* | phosphofructokinase, muscle | 5' | CGTCCCTGGGTCAGACTTCAG |
|  | 18642 | 3' | CAGACTGCTTGATTCGGTCACA |
|  |  | probe | CTGACACAGCACTGAACACCATCTGCAC |
| *Pln* | phospholamban | 5' | CAGGAGAGCCTCCACTATTGAAA |
|  | 18821 | 3' | GATGAGGCAGAAATTGATAAATAGGTT |
|  |  | probe | CCTCAGCAAGCACGTCAGAATCTCCA |
| *Ppargc1a*  *(Pgc-1a)* | peroxisome proliferative activated receptor, gamma, coactivator 1 alpha 19017 | 5'  3'  probe | AGCGACCAATCGGAAATCAT  GCAAGTTTGCCTCATTCTCTTCA  TCCAACCAGTACAACAATGAGCCTGCG |
| *Rn18s* | 18S ribosomal RNA | 5' | TGGTTGCAAAGCTGAAACTTAAAG |
| *(18s)* | 19791 | 3' | AGTCAAATTAAGCCGCAGGC |
|  |  | probe | CCTGGTGGTGCCCTTCCGTCA |
| *Ryr2* | ryanodine receptor 2, cardiac | 5' | CAGCAGCCCCCACAGG |
|  | 20191 | 3' | TTCCATGTAGCCGCTGCTC |
|  |  | probe | TCATTGCGGTTCACTATGTCCTGGAGG |
| *Scn4b* | sodium channel, type IV, beta | 5'  3' | GAACCGAGGCAATACTCA  CGATGAGCCGTTAATAGC |
|  | 399548 | probe | CCACAGATACCTCCAACGACAGG |
| *Scn5a* | sodium channel, voltage-gated, type V, alpha | 5'  3' | CAGCAGCTTCCGTAGGTTCAC  TTTCAGCCATGCGCTTCTC |
|  | 20271 | probe | TGGCCGCCAGTGACTCCCG |
| *Slc2a4*  *(Glut4)* | solute carrier family 2 (facilitated glucose transporter), member 4 20528 | 5'  3'  probe | CCTGGCCGGCATGTGT  GAGACATAGCTCATGGCTGGAA  CCATCTTGATGACCGTGGCTCTGCT |
| *Slc8a1*  *(Ncx)* | solute carrier family 8 (sodium/calcium exchanger), member 1 20541 | 5'  3'  probe | TTGTTTTCCCATGTTGACCATATAA  GAGCCAGTACATTCAGTGGTTTCA  TGCAGATACAGAGGCAGAAACAGGAGGAA |
| *Slc27a1* | solute carrier family 27 (fatty acid transporter), member 1 | 5'  3' | TCTACGGGTTGACGGTGGTACT  TCAGCAGGTAGCGGCAGATT |
| *(Fatp1)* | 26457 | probe | CAAGAAGTTCTCCGCCAGCCGCTT |
| *Tfam* | transcription factor A, mitochondrial | 5'  3' | TTCGTTACGACAATGAAATGAAGTC  TCGACGGATGAGATCACTTCG |
|  | 21780 | probe | TGGGAAGAGCAGATGGCTGAAGTTGG |

**Supplementary Table S2. Boltzmann fitting result (L-type Ca^2+^ - current).**

|  | WT | TG | p-Value |
| --- | --- | --- | --- |
| Activation | | | |
| *V½*, mV | -3.2 ± 0.7 | -3.0 ± 1.1 | >0.05 |
| Slope factor | 4.3 ± 0.3 | 3.6 ± 0.2 | >0.05 |
| Inactivation | | | |
| *V½*, mV | -14.2 ± 0.4 | -13.1 ± 0.9 | >0.05 |
| Slope factor | 4.3 ± 0.2 | 4.3 ± 0.2 | >0.05 |

**Supplementary Table S3. Boltzmann and exponential fitting results (Na^+^ - current).**

|  | WT | TG | p-Value |
| --- | --- | --- | --- |
| Activation | | | |
| *V½*, mV | -51.7± 0.6 | -48.9 ± 0.6 | 0.003 |
| Slope factor | 2.3 ± 0.2 | 2.5 ± 0.2 | >0.05 |
| Inactivation | | | |
| *V½*, mV | -80.3 ± 0.4 | -78.9 ± 0.3 | 0.008 |
| Slope factor | 4.4 ± 0.1 | 4.5 ± 0.1 | >0.05 |
| Recovery from inactivation | | | |
| Time constant | -0.31 ± 0.03 | -0.29 ± 0.04 | >0.05 |

1. **Supplemental References**

Belke, D. D., T. S. Larsen, G. D. Lopaschuk and D. L. Severson (1999). Glucose and fatty acid metabolism in the isolated working mouse heart *Am J Physiol* **277**(4 Pt 2): R1210-1217.

Bry, M., R. Kivela, T. Holopainen, A. Anisimov, T. Tammela, J. Soronen, et al. (2010). Vascular endothelial growth factor-B acts as a coronary growth factor in transgenic rats without inducing angiogenesis, vascular leak, or inflammation. *Circulation* **122**(17): 1725-1733, doi: 10.1161/CIRCULATIONAHA.110.957332.

Koivumaki, J. T., T. Korhonen, J. Takalo, M. Weckstrom and P. Tavi (2009). Regulation of excitation-contraction coupling in mouse cardiac myocytes: integrative analysis with mathematical modelling. *BMC Physiol* **9**: 16, doi: 10.1186/1472-6793-9-16.

Merentie, M., J. A. Lipponen, M. Hedman, A. Hedman, J. Hartikainen, J. Huusko, et al. (2015). Mouse ECG findings in aging, with conduction system affecting drugs and in cardiac pathologies: Development and validation of ECG analysis algorithm in mice. *Physiol Rep* **3**(12), doi: 10.14814/phy2.12639.

Mitchell, G. F., A. Jeron and G. Koren (1998). Measurement of heart rate and Q-T interval in the conscious mouse. *Am J Physiol* **274**(3 Pt 2): H747-751.

Rivard, K., V. Trepanier-Boulay, H. Rindt and C. Fiset (2009). Electrical remodeling in a transgenic mouse model of alpha1B-adrenergic receptor overexpression *Am J Physiol Heart Circ Physiol* **296**(3): H704-718, doi: 10.1152/ajpheart.00337.2008.

Sato, P. Y., H. Musa, W. Coombs, G. Guerrero-Serna, G. A. Patino, S. M. Taffet, et al. (2009). Loss of plakophilin-2 expression leads to decreased sodium current and slower conduction velocity in cultured cardiac myocytes ina. *Circ Res* **105**(6): 523-526, doi: 10.1161/CIRCRESAHA.109.201418.

Sossalla, S., B. Kallmeyer, S. Wagner, M. Mazur, U. Maurer, K. Toischer, et al. (2010). Altered Na(+) currents in atrial fibrillation effects of ranolazine on arrhythmias and contractility in human atrial myocardium. *J Am Coll Cardiol* **55**(21): 2330-2342, doi: 10.1016/j.jacc.2009.12.055.

Xu, L., X. Y. Li, Y. Liu, H. T. Li, J. Chen, X. Y. Li, et al. (2011). The mechanisms underlying ICa heterogeneity across murine left ventricle *Mol Cell Biochem* **352**(1-2): 239-246, doi: 10.1007/s11010-011-0759-8.

Yang, Z., W. Shen, J. N. Rottman, J. P. Wikswo and K. T. Murray (2005). Rapid stimulation causes electrical remodeling in cultured atrial myocytes *J Mol Cell Cardiol* **38**(2): 299-308, doi: 10.1016/j.yjmcc.2004.11.015.
